# Development of a fully automatic deep learning system for L3 selection and body composition assessment on computed tomography

**DOI:** 10.1038/s41598-021-00161-5

**Published:** 2021-11-04

**Authors:** Jiyeon Ha, Taeyong Park, Hong-Kyu Kim, Youngbin Shin, Yousun Ko, Dong Wook Kim, Yu Sub Sung, Jiwoo Lee, Su Jung Ham, Seungwoo Khang, Heeryeol Jeong, Kyoyeong Koo, Jeongjin Lee, Kyung Won Kim

**Affiliations:** 1 Department of Radiology and Research Institute of Radiology, Asan Medical Center, University of Ulsan College of Medicine, 88 Olympic-ro, 43-gil, Songpa-gu, Seoul, 05505 Korea; 2 Department of Radiology and Research Institute of Radiology, University of Ulsan College of Medicine, Seoul, Korea; 3 Health Screening and Promotion Center, Asan Medical Center, University of Ulsan College of Medicine, Seoul, Korea; 4 Biomedical Research Center, Asan Institute for Life Sciences, Asan Medical Center, Seoul, Korea; 5 Clinical Research Center, Asan Medical Center, Seoul, Korea; 6 Department of Convergence Medicine, University of Ulsan College of Medicine, Seoul, Korea; 7 School of Computer Science and Engineering, Soongsil University, Seoul, Korea

**Keywords:** Computational biology and bioinformatics, Biomarkers, Diseases

## Abstract

As sarcopenia research has been gaining emphasis, the need for quantification of abdominal muscle on computed tomography (CT) is increasing. Thus, a fully automated system to select L3 slice and segment muscle in an end-to-end manner is demanded. We aimed to develop a deep learning model (DLM) to select the L3 slice with consideration of anatomic variations and to segment cross-sectional areas (CSAs) of abdominal muscle and fat. Our DLM, named L3SEG-net, was composed of a YOLOv3-based algorithm for selecting the L3 slice and a fully convolutional network (FCN)-based algorithm for segmentation. The YOLOv3-based algorithm was developed via supervised learning using a training dataset (n = 922), and the FCN-based algorithm was transferred from prior work. Our L3SEG-net was validated with internal (n = 496) and external validation (n = 586) datasets. Ground truth L3 level CT slice and anatomic variation were identified by a board-certified radiologist. L3 slice selection accuracy was evaluated by the distance difference between ground truths and DLM-derived results. Technical success for L3 slice selection was defined when the distance difference was < 10 mm. Overall segmentation accuracy was evaluated by CSA error and DSC value. The influence of anatomic variations on DLM performance was evaluated. In the internal and external validation datasets, the accuracy of automatic L3 slice selection was high, with mean distance differences of 3.7 ± 8.4 mm and 4.1 ± 8.3 mm, respectively, and with technical success rates of 93.1% and 92.3%, respectively. However, in the subgroup analysis of anatomic variations, the L3 slice selection accuracy decreased, with distance differences of 12.4 ± 15.4 mm and 12.1 ± 14.6 mm, respectively, and with technical success rates of 67.2% and 67.9%, respectively. The overall segmentation accuracy of abdominal muscle areas was excellent regardless of anatomic variation, with CSA errors of 1.38–3.10 cm^2^. A fully automatic system was developed for the selection of an exact axial CT slice at the L3 vertebral level and the segmentation of abdominal muscle areas.

## Introduction

The segmentation of muscle and fat areas on abdominal computed tomography (CT) has gained huge emphasis in the last decade, as sarcopenia research has been growing rapidly. According to the revised European Working Group on Sarcopenia in Older People (EWGSOP2)^1^, the muscle area on CT measured at the third lumbar vertebral level is used as a representative value because it can reflect the whole-body muscle mass^2–5^.

Recent studies reported the influence of body muscle and fat mass on the prognosis of various diseases and recovery from surgery. Visceral fat was suggested as the main factor for metabolic syndrome, as it was influent to insulin regulation^6, 7^. And larger visceral fat mass increased the risk of cardiovascular disease^8^. Sarcopenia has been reported as a potential biomarker to the prognosis of various cancer and recovery from various surgery^9–12^. Therefore, the necessity to measure muscle and fat areas on CT has increased rapidly^13^, increasing the demand for automatic muscle and fat measurement technologies such as the deep learning model (DLM). Accordingly, there have been several previous studies that developed automatic segmentation for body composition analysis using DLM^14–20^, and some of them are commercially available^21^. These new automatic segmentation methods can reduce the time to measure abdominal muscle and fat areas to some degree. Still, these techniques have required manual selection of L3 slice CT images, which might be the greatest hurdle to achieve fully automatic body composition measurements. In general, it takes several minutes (around three minutes) to find L3 slice level on abdominal CT even by experts. The time spent was defined to include opening the software, importing the prepared CT images, finding the L3 inferior endplate level, and segmenting the abdominal muscle according to a prior study^22^.

So far, only a few studies have attempted to develop a fully automatic technique for L3 slice selection and muscle segmentation^23, 24^. However, these studies have not been clinically validated well; especially, it is unclear whether or not these studies have developed automatic L3 slice selection techniques with consideration of thoracolumbar/lumbosacral variations. Thoracolumbar/lumbosacral variations may occur in around 20% (4–30%) of normal population^25, 26^. Therefore, developing a DLM-based automatic L3 slice selection technique requires training data with full consideration of anatomic variations.

The primary objective of this study was to develop a DLM to automatically select L3 slices on abdominal CT scans and then automatically segment areas of the abdominal muscle, visceral fat, and subcutaneous fat. The secondary objective was to validate the accuracy of DLM to select L3 slices with consideration of anatomic variations. The third objective was to validate the segmentation accuracy of DLM to measure muscle and fat areas at the L3 level.

## Materials and methods

This study was approved by the institutional review boards of Asan Medical Center (AMC), Kyung Hee University Hospital (KHUH), Ajou University Hospital (AUH), and Ulsan University Hospital (UUH). The informed consent requirement was waived by the institutional review board of Asan Medical Center. The research has been performed following the Declaration of Helsinki and all experimental protocols were carried according to the experimental guideline and regulations of the Asan Institute for Life Sciences.

This article reports on and complies with the methods and terms described in the most recently published guidance on reading literature about machine learning for medical applications^27^.

### Data acquisition: study subjects

**Figure 1:**
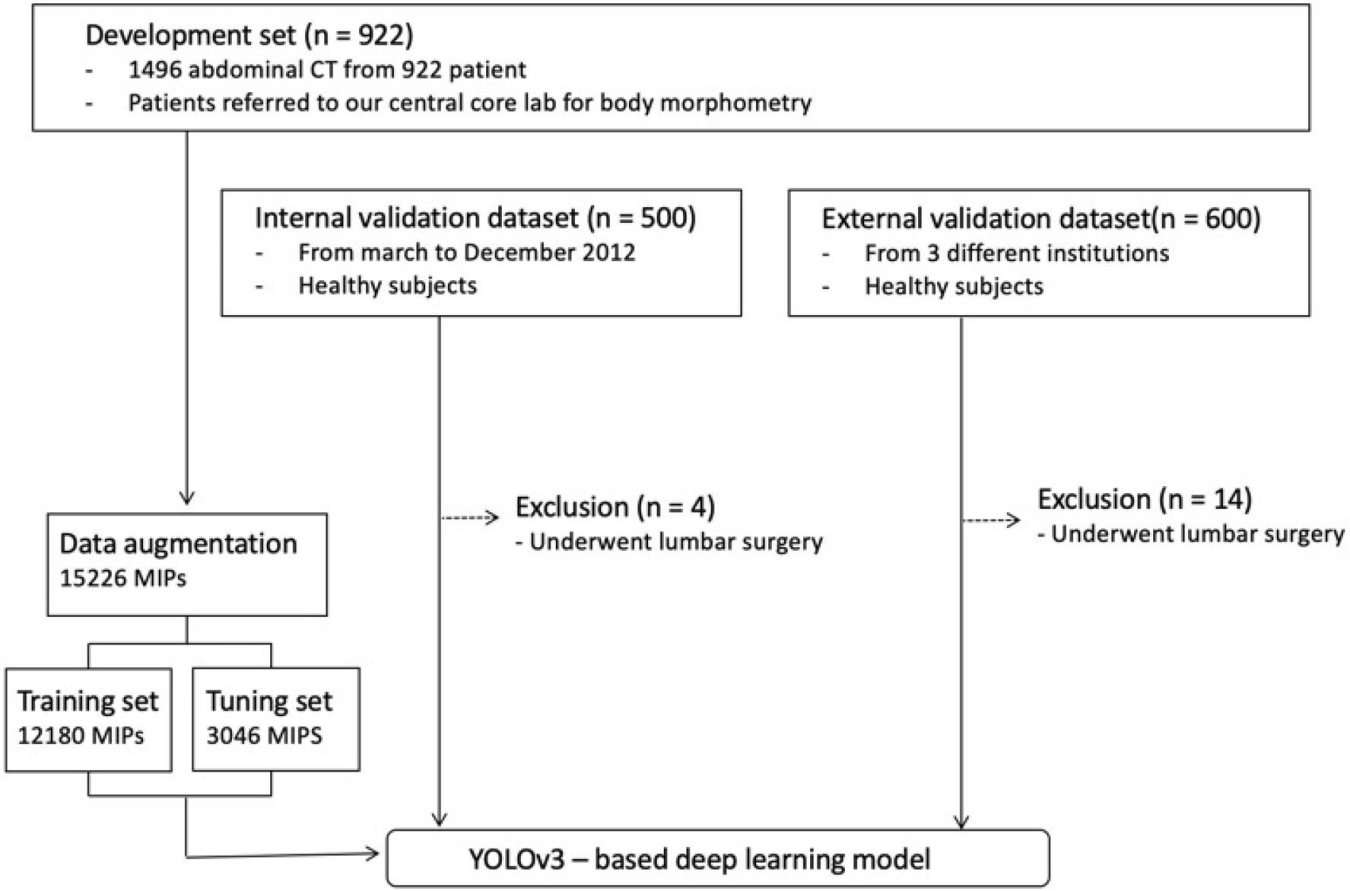
An overview of dataset composition.

The datasets used for this study were as follows: (1) development dataset used for developing the DLM, which was further split into the training set and tuning set; (2) validation dataset for independent testing of model performance, including an internal validation set and an external validation set. An overview of dataset composition is described in Fig. 1.

The development dataset was composed of 922 patients (560 men and 362 women; mean age, 54.4 ± 14.0 years), with 1496 abdominal CT images obtained from the AMC. The development dataset was used in our previous study^14^. The development dataset included patients with various diseases and healthy subjects, as follows: various cancer patients who underwent APCT for diagnostic purposes or treatment monitoring, healthy subjects who underwent APCT as routine clinical practice for organ donation, and patients with severe inflammation resulting in sepsis. Adult patients were only included in the development dataset. We tried to organize the dataset to reflect the real clinical practice by including both patients with various diseases and healthy subjects. To identify anatomic variations accurately, we also obtained chest CT scans in 910 patients.

The internal validation set was composed of 500 healthy subjects who had both chest CT and abdominal CT scans acquired in our institution from March through December 2012. Four subjects who underwent interbody lumbar vertebra fusion surgery were excluded, and a total of 496 subjects with 496 CT scans were used for validation (301 men and 195 women; mean age, 53.7 ± 8.7 years). The external validation dataset included 600 patients who had both chest and abdominal CT scans, acquired between September 2011 and March 2019 from three other institutions (KHUH, AUH, and UUH). A total of 586 patients were included after excluding 14 subjects who underwent lumbar interbody fusion surgery (347 men and 239 women; mean age, 58.5 ± 12.3). The clinical characteristics of subjects included in the validation dataset are summarized in Table 1. Healthy subjects conducted CT for evaluation for organ donation including liver and kidney. An abdominal CT scan is a part of routine clinical management for potential liver or kidney donors. The healthy subject who underwent CT scans for benign lesions were also included in internal and external validation groups. Ultrasonography is a screening method widely used due to absence of radiation hazards. If a focal lesion detected on the ultrasonography, CT scan is usually conducted for further characterization of the lesion in clinical practice.

**Table 1:** Subject characteristics of internal and external validation cohorts.

Characteristics	Development dataset	Internal validation dataset	External validation dataset
Number of subjects	922	496	586
Age (years)	54.4 ± 14.0	53.7 ± 8.7	58.5 ± 12.3
Female (%, female:male)	39.3% (362:560)	39.3% (195:301)	40.8% (239:347)
**Anatomic variation**			
Normal anatomy group	807 (87.5%)	438 (88.3%)	505 (86.2%)
Anatomic variants group	115 (12.5%)	58 (11.7%)	81 (13.8%)
Thoracolumbar variant	48 (5.2%)	20 (4.0%)	26 (4.4%)
Lumbosacral variant	43 (4.7%)	29 (5.8%)	43 (7.3%)
Numeric variant	12 (1.3%)	4 (1.4%)	7 (1.2%)
Combined variant	12 (1.3%)	5 (1.7%)	5 (0.9%)
Institution	AMC	AMC	UUH, KHUH, AUH
**Underlying disease (n)**			
None (healthy)	87	496	586
Gastric cancer	436	0	0
Sepsis	245	0	0
Pancreatic cancer	154	0	0

*AMC* Asan Medical Center**,** *AUH* Ajou University Hospital**,** *KHUH* Kyung Hee University Hospital**,** *UUH* Ulsan University Hospital.

CT scanners from various manufacturers with different acquisition protocols were used in all datasets, so that we trained a universal deep learning model and validate its generalizability across different datasets. Detailed specifications of the abdominal CT acquisition are summarized in Supplementary Table 1.

### Generation of the ground truth

For each CT scan, the axial CT slice number of the third lumbar vertebra inferior endplate was annotated, and the lumbar vertebral anatomic variant was identified by a board-certified radiologist (J.H.) and double-checked by another radiologist (K.W.K.). In most cases, we counted the number of thoracolumbar spines and ribs in chest CT and abdominal CT scans to identify the anatomic variations accurately. Disagreement was resolved by reaching a consensus through discussion.

**Figure 2:**
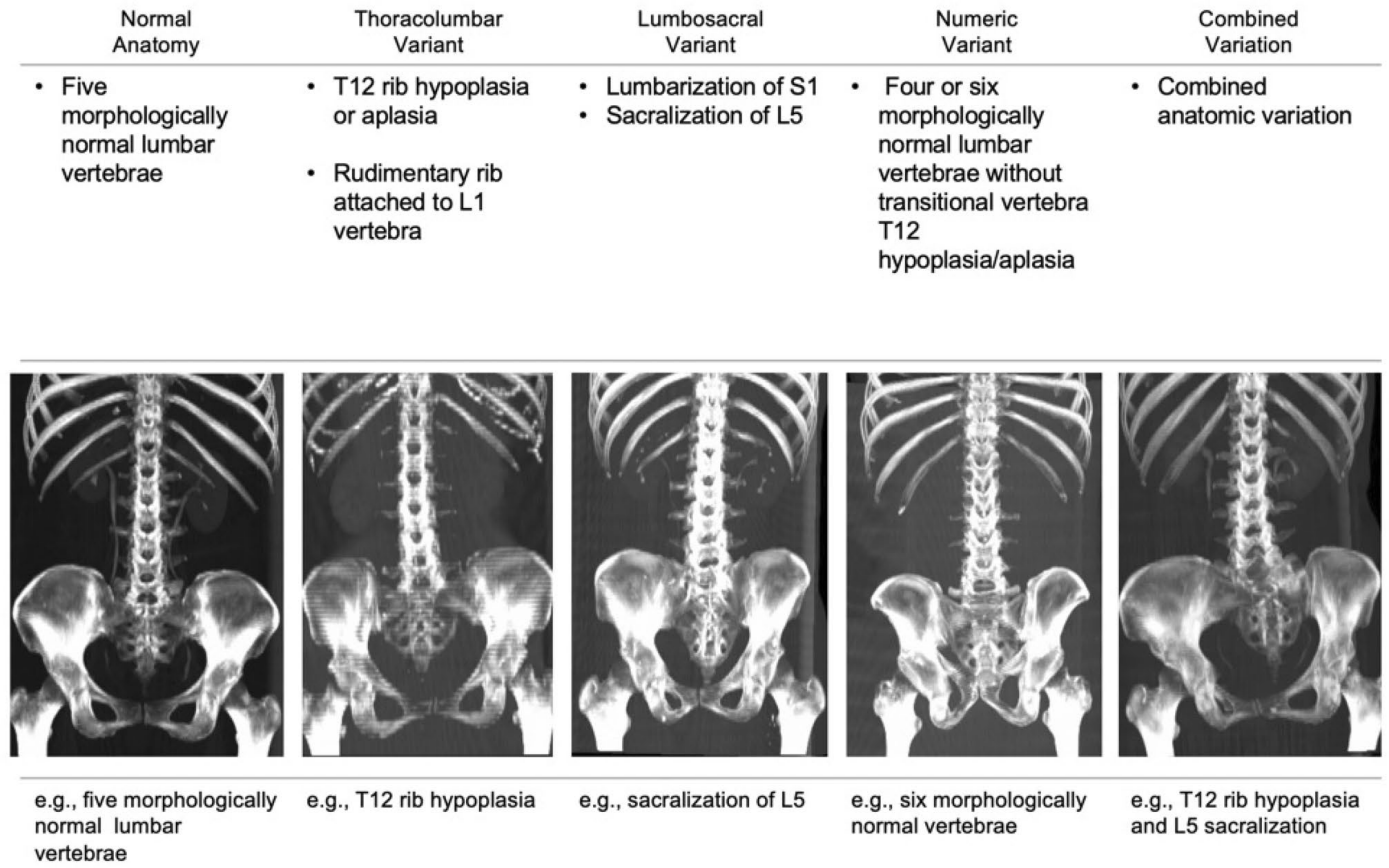
Anatomic lumbar spine variants. Examples of normal, thoracolumbar, lumbosacral, numeric, and combined variations are presented.

According to the vertebral anatomy, patients were divided into the normal anatomy group and the anatomic variant group. The anatomic variants were categorized into four subgroups as follows: (1) thoracolumbar variant (twelfth rib aplasia/hypoplasia or rudimentary rib of L1), (2) lumbosacral variant (lumbarization or sacralization), (3) numeric variant (four or six lumbar vertebrae without transitional vertebra), and (4) combination of two different variants^28–30^. Figure 2 summarized the type of anatomic variant. Morphologically normal ribs were defined as a pair of ribs that were 3.8 cm in length or more and originated from the facet between the pedicle and vertebral body. Lumbosacral transitional vertebrae were identified based on the criteria described by Castellvi et al. in 1984^31^. Lumbar vertebrae without rudimentary or normal ribs and showing normal transverse processes were regarded as morphologically normal lumbar vertebrae.

An expert image analyst (S.J.H., 11 years experience in image analysis) manually generated the GT segmentation map for skeletal muscle area (SMA), visceral fat area (Vfat), and subcutaneous fat area (Sfat). The segmentation map was double-checked by a supervising radiologist (K.W.K.).

### Deep learning model development

Our DLM was composed of two algorithms, as follows: (1) a YOLOv3-based algorithm for selecting the L3 slice and (2) a fully convolutional network (FCN)-based algorithm for segmentation. These two algorithms were packaged in a DLM toolkit, named L3SEG-net.

Several preprocessing steps were used to generate input data to increase the effective dataset size and improve overfitting and accuracy. Data augmentation was performed to generate 15,226 maximum intensity projection (MIP) images from 1496 CT scans. Of these, 12,180 MIP images were used as a training set, and 3046 images were used as a tuning set. CT scans were converted to MIP images to feed the DLM because MIP images can give various information with a single image including morphologic features of each vertebral body, location, and anatomic variation.

### YOLOv3-based L3 slice selection algorithm

**Figure 3:**
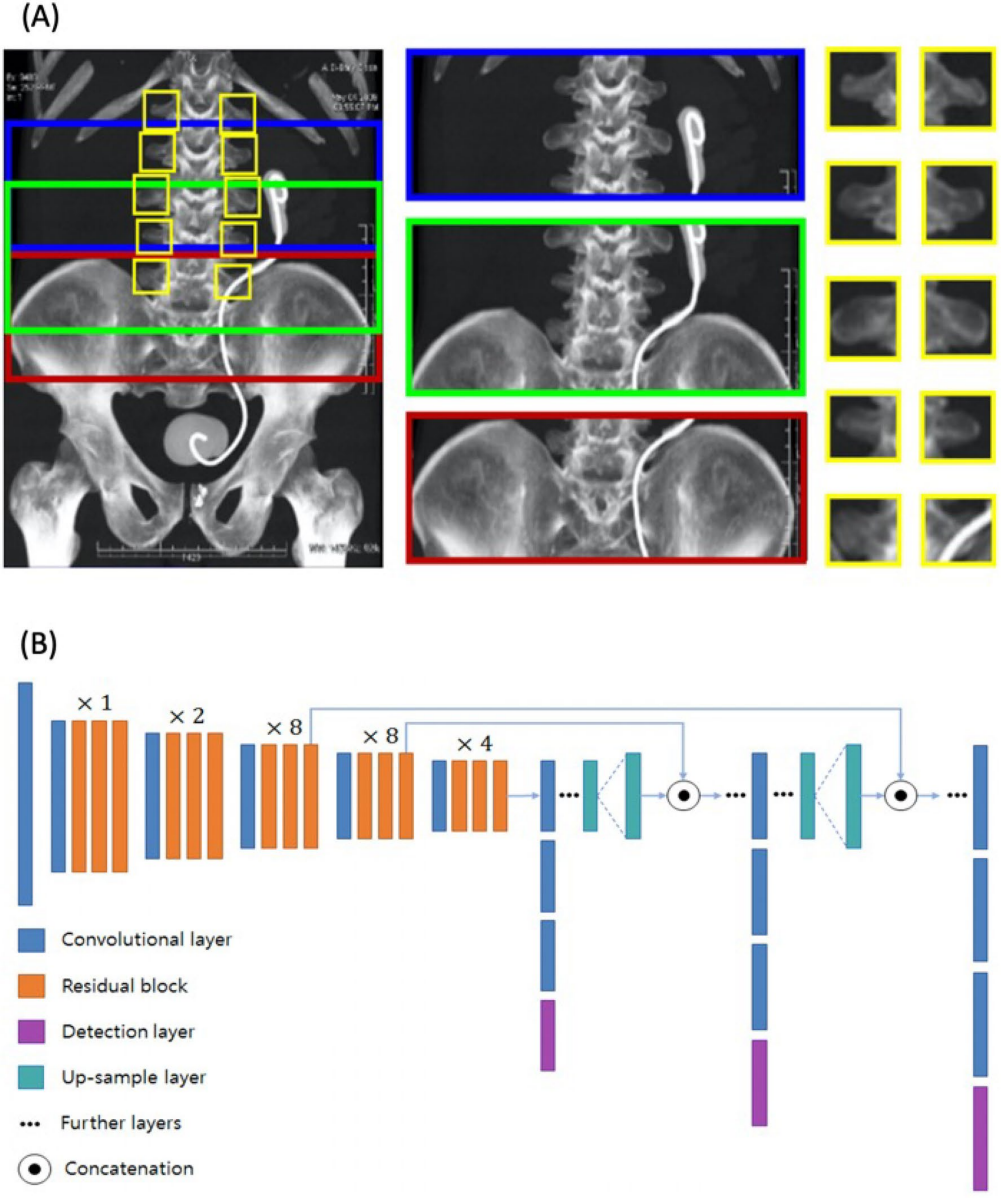
Example of multiple bounding boxes for the training of the YOLOv3-based model and architecture of our YOLOv3-based network. Multiple bounding boxes were generated in the maximum intensity projection images based on the following prerequisites as illustrated in **(A)**: (1) the L4 vertebra was located at the iliac crest level, (2) the L3 vertebra was located superiorly to the L4 vertebra, (3) the morphologies of the lumbar vertebrae were the same. The YOLOv3-based model used an objectness score for each bounding box obtained from logistic regression to predict the width and height of the box as well as its location relative to grid cell. The sum of the squared error loss was used to train the model for minimizing differences between the ground-truth object and the bounding box. Any error between the bounding box over the ground-truth object was incurred for both classification and detection loss. Our model extracted features of the bounding boxes using the network architecture illustrated in **(B)**. Our network architecture used successive 3 × 3 and 1 × 1 convolution layers and a set of residual blocks with shortcut connections. A total of 53 convolutional layers were formed like Darknet-53. YOLOv3 predicted boxes at three different scales to support detection on varying scales.

A YOLOv3-based algorithm was adopted because YOLOv3 can detect objects and extract features more efficiently than conventional convolutional neural networks, accomplished via object detection and classification^32^. Our YOLOv3-based algorithm generated multiple bounding boxes to extract features from MIP images using a concept similar to feature pyramid networks^33^. The L3 endplate was localized in a MIP image using extracted features of multiple bounding boxes and their relative coordinates. Network architecture and an example of bounding boxes are shown in Fig. 3.

### FCN-based segmentation algorithm

Our FCN-based algorithm for automatic segmentation is described elsewhere^14^. We added several post-processing steps to separate the intramuscular adipose tissue from the SMA based on Hounsfield units. The network architecture of our FCN-based algorithm is illustrated in Supplementary Fig. 1. Our FCN-based segmentation algorithm yielded cross-sectional areas (CSAs) of SMA, Vfat, and Sfat in cm^2^ at the selected L3 slice CT images. Currently, the FCN-based segmentation algorithm is available as a web-based iAID toolkit^34^.

### Validation of deep learning model

#### Accuracy of automatic L3 slice selection

In both internal and external validation cohorts, the L3 slice selection accuracy of the YOLOv3-based algorithm was evaluated by the absolute distance difference between the GT and the DLM-derived CT slice. The differences in CT slice numbers between the GT and the DLM-derived results were calculated and multiplied by slice thickness to generate the actual distance difference in millimeters. Technical success was defined when the distance difference between the GT and the DLM-derived results was less than 10 mm (Supplementary Fig. 2). The distance difference and technical success were separately evaluated in the normal anatomy group and anatomic variant group.

#### Segmentation accuracy of the DLM

In both internal and external validation datasets, the CSA error was calculated to evaluate the accuracy of the DLM-derived segmentation, which is a result of a combination of the YOLOv3-based L3 slice selection and the FCN-based segmentation of abdominal muscle and fat. The CSA error is a standardized percentage difference in measured areas of muscle and fat between the GT values and the DLM-derived values. Thus, a low CSA error implies a high segmentation accuracy. The CSA error was calculated using the following equation:

$${\text{CSA}}\;{\text{error}}\;(\% ) = \frac{{|{\text{ground}}\;{\text{truth}}\;{\text{CSA}} - {\text{DLMCSA}}|}}{{{\text{ground}}\;{\text{truth}}\;{\text{CSA}}}} \times 100$$

In subjects with concordant L3 levels, i.e., identical CT slice numbers from both the GT and the DLM-derived results. The Dice similarity coefficient (DSC) was also used to evaluate the segmentation accuracy of our DLM. DSC is an index of spatial overlap ranging from 0 to 1. Completely overlapped area shows DSC value of 1, whereas no overlapped area shows DSC value 0. DSC was calculated according to the equation described below:

$${\text{DSC}} = \frac{{2 \times \left| {{\text{ground}}\;{\text{truth}} \cap {\text{FCN}}} \right|}}{{\left| {{\text{ground}}\;{\text{truth}}} \right| + \left| {{\text{FCN}}} \right|}}$$

$${\text{DSC}} = \frac{{2 \times TP{_\text{P}}}}{{2 \times TP{_\text{P}} + FP{_\text{V}} + FN{_\text{V}}}}$$

*TP*_P_ denotes number of pixels which is correctly included, in both GT and DLM driven result. *FP*_v_ means number of pixels included in DLM driven result but not in GT. *FN*_v_ represents number of pixels included in GT but not in DLM driven results.

### Subgroup analysis according to anatomic variation

The influence of anatomic variation on the performance of the DLM when selecting the L3 slice and segmenting muscle and fat areas were explored by subgroup analysis. The whole validation cohort, i.e., combined internal and external validation cohorts, was divided according to spinal anatomic variations. The accuracy of L3 slice selection and the segmentation accuracy of the DLM was compared between these subgroups.

### Statistical analysis

The average values of distance differences between the GT and the DLM-derived L3 slices were compared between the normal anatomy group and the anatomic variant group using a Student t-test. The technical success rate, i.e., the percentage of subjects with technical success among all subjects, was compared between the normal anatomy group and the anatomic variant group using the chi-square test.

The average CSA values of SMA, Sfat, and Vfat were compared between the GT and the DLM-derived results using paired t-tests. The CSA errors were compared between subjects with technical success and subjects with technical failure in the internal and external validation datasets.

Agreement in the measured CSAs of muscle and fat between GT values and DLM-derived values was evaluated with Bland–Altman plots with 95% limits of agreement. R version 3.6.3 (R Foundation for Statistical Computing, Vienna, Austria), MedCalc 12.7.0 (MedCalc Software, Mariakerke, Belgium) were used for statistical analysis. A p-value < 0.05 was regarded as statistically significant.

## Results

### Accuracy of automatic L3 slice selection

**Figure 4:**
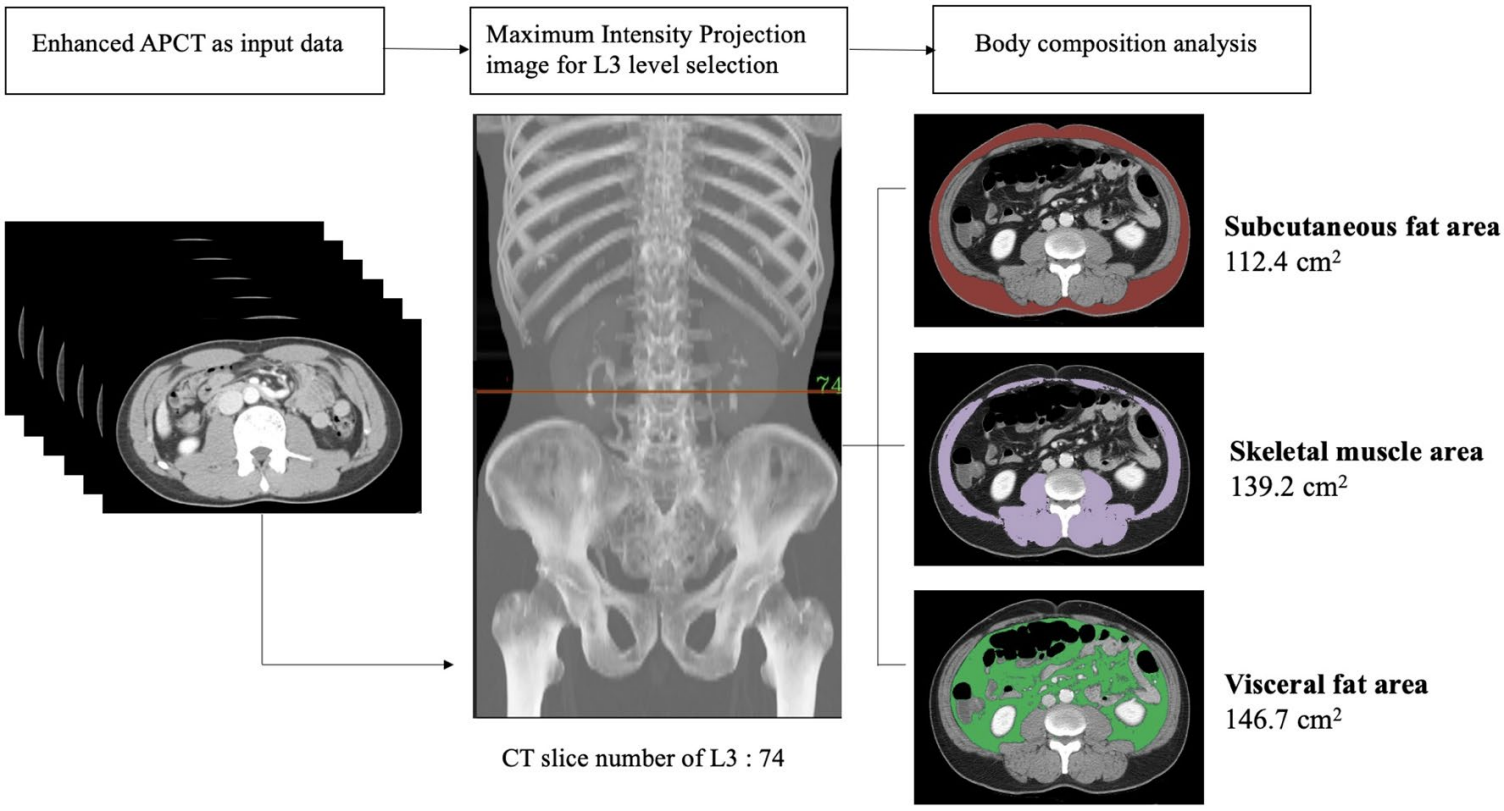
Example of L3 level selection and body composition analysis using L3SEG-net. APCT was converted to a MIP image. L3 level was selected by YOLOv3 based selection algorithm and selected CT slice number was transferred to FCN based segmentation algorithm. The final output of L3SEG-net was areas of each composition element including subcutaneous fat area, skeletal muscle area, and visceral fat area. *APCT* abdominopelvic computed tomography, *MIP* maximal intensity projection.

**Figure 5:**
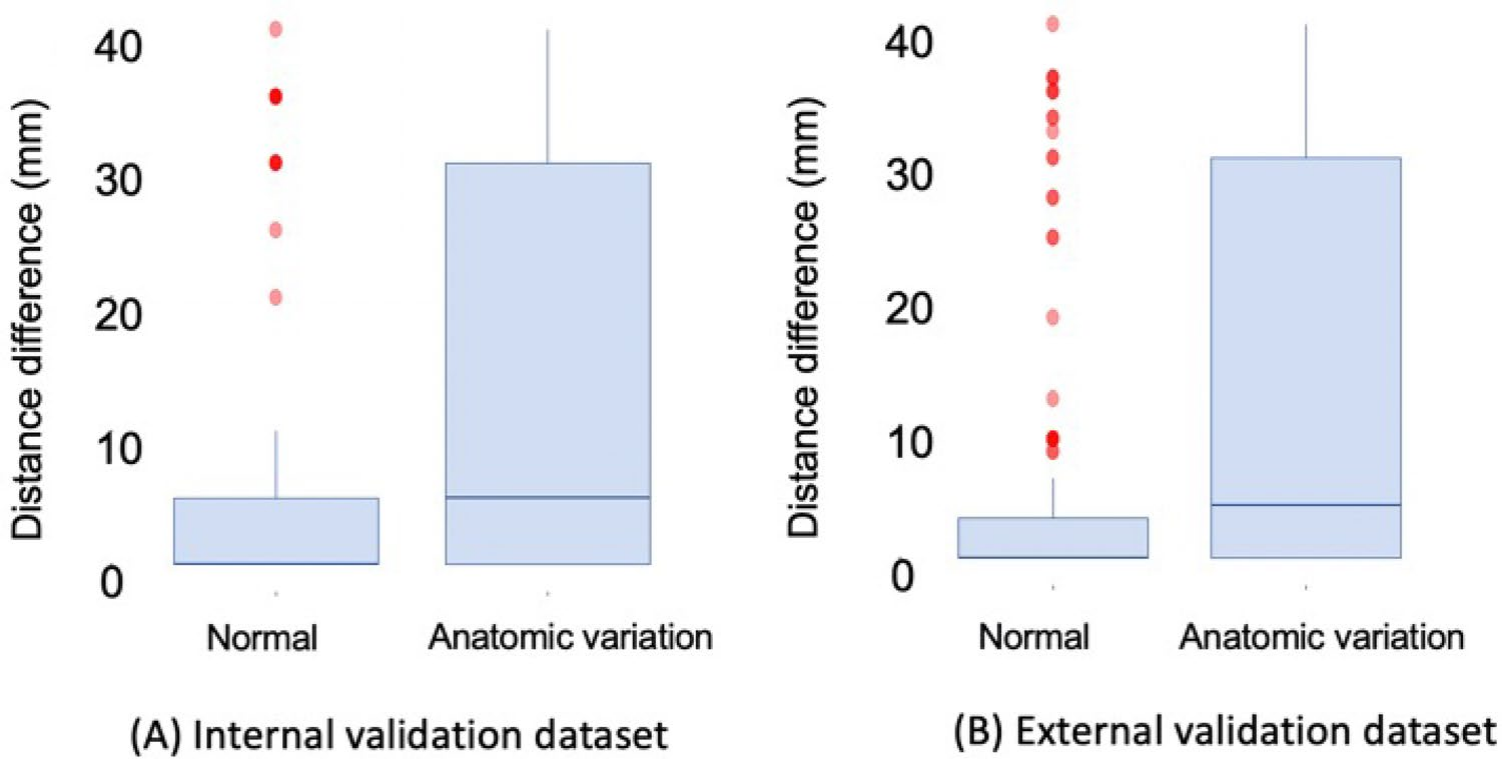
Box plots of distance difference between ground truth and deep learning model (DLM) derived results in **(A)** internal validation dataset and **(B)** external validation cohorts. The mean differences between the ground truth and the DLM-derived results were 3.7 mm ± 8.4 and 4.1 mm ± 8.3 for the internal, and external validation cohorts, respectively.

The outline of L3 slice level selection and segmentation of body composition were presented in Fig. 4. The accuracy of the YOLOv3-based algorithm for automatic L3 slice selection in the internal and external validation datasets is summarized in Fig. 5. The mean distance differences between the GT and the DLM-derived L3 slices were 3.7 ± 8.4 mm and 4.1 ± 8.3 mm for the internal and external validation cohorts, respectively. Subjects with normal spinal anatomy yielded smaller distance differences than those with anatomic variants in the internal (2.5 ± 6.1 vs.12.4 ± 15.4 mm, p < 0.001) and external (2.8 ± 5.9 vs. 12.1 ± 14.6 mm, p < 0.001) validation sets. The maximum distance difference was 40 mm, equivalent to the height of a vertebral body.

Technical success was achieved for 93.1% (463/496) and 92.3% (541/586) of subjects in the internal and external validation datasets, respectively. The normal anatomy group yielded higher technical success rates than the anatomic variant group in the internal (96.6% vs. 67.2%, p < 0.001) and external (96.2% vs. 67.9%, p < 0.001) validation datasets.

### Segmentation accuracy of DLM-derived abdominal muscle and fat areas

The average CSAs of SMA, Sfat, and Vfat derived from GT and DLM are presented in Table 2. In all subjects of internal and external validation datasets, there were no significant differences in CSAs between the GT and DLM-derived measurements in SMA, Sfat, and Vfat (p > 0.05 for all comparisons). Even for subjects with technical failure of L3 slice selection, the CSAs did not differ significantly between the GT and the DLM-derived measurements (p > 0.05 for all comparisons).

**Table 2:** Cross-sectional area segmentation using the ground truth-derived and DLM-derived levels.

Parameter	Internal validation dataset			External validation dataset		
	SMA	Sfat	Vfat	SMA	Sfat	Vfat
**All subjects (n = 1082)**						
CSA from GT (cm^2^)	140.88 ± 34.53	140.90 ± 56.71	114.53 ± 65.05	132.76 ± 31.25	133.15 ± 62.16	110.59 ± 64.29
CSA from DLM (cm^2^)	140.53 ± 34.20	141.98 ± 56.60	115.93 ± 65.40	130.07 ± 31.07	135.54 ± 62.64	110.72 ± 65.19
p value*	0.874	0.764	0.736	0.139	0.492	0.973
CSA error (%)	1.38 ± 1.46	3.51 ± 5.41	4.00 ± 6.35	3.10 ± 2.85	4.54 ± 6.34	4.26 ± 6.47
**Subjects with technical success (n = 1004)**						
CSA from GT (cm^2^)	141.20 ± 34.46	138.85 ± 55.86	112.42 ± 64.73	132.75 ± 31.15	133.99 ± 62.82	110.88 ± 64.18
CSA from DLM (cm^2^)	140.87 ± 34.06	140.47 ± 55.72	114.11 ± 64.95	130.14 ± 31.00	136.73 ± 63.15	111.13 ± 65.06
p value*	0.883	0.659	0.692	0.167	0.474	0.950
CSA error (%)	1.22 ± 1.08	2.31 ± 2.21	2.97 ± 3.21	2.86 ± 2.57	3.39 ± 2.78	3.36 ± 4.68
**Subjects with technical failure (n = 78)**						
CSA from GT (cm^2^)	136.33 ± 35.18	169.78 ± 60.54	144.23 ± 62.27	132.97 ± 32.3	123.03 ± 52.30	107.10 ± 65.61
CSA from DLM (cm^2^)	135.77 ± 35.80	163.24 ± 64.41	141.53 ± 66.37	129.21 ± 31.93	122.66 ± 54.42	105.78 ± 66.50
p value*	0.949	0.672	0.865	0.579	0.974	0.924
CSA error (%)	3.68 ± 3.19	20.42 ± 8.14	18.37 ± 15.68	6.01 ± 4.18	18.28 ± 15.16	15.06 ± 12.56
p value^§^	< 0.01	< 0.01	< 0.01	< 0.01	< 0.01	< 0.01

Data are presented as mean ± standard deviation.

*The p-value is calculated from Student t-test comparing the GT CSA and the CSA determined using the DLM.

^§^The p-value is calculated from Student t-test comparing CSA errors between subjects with technical success and subjects with technical failure.

*CSA* cross-sectional area, *DLM* deep learning model, *GT* ground truth, *Sfat* subcutaneous fat area, *SMA* skeletal muscle area, *Vfat* visceral fat area.

The average CSA errors of SMA, Sfat, and Vfat in all subjects of the internal and external validation datasets ranged from 1.38% to 4.54% (Table 2), indicative of excellent segmentation accuracy of the DLM. When we divided them into subjects with technical success and subjects with technical failure in terms of L3 slice selection, the average CSA errors of subjects with technical failure were higher than those with technical success in both the internal and external validation groups (p < 0.05 for all comparisons). However, such CSA errors in subjects with technical failure were relatively small in the SMA, compared with the Sfat and Vfat in both the internal and external validation datasets.

**Figure 6:**
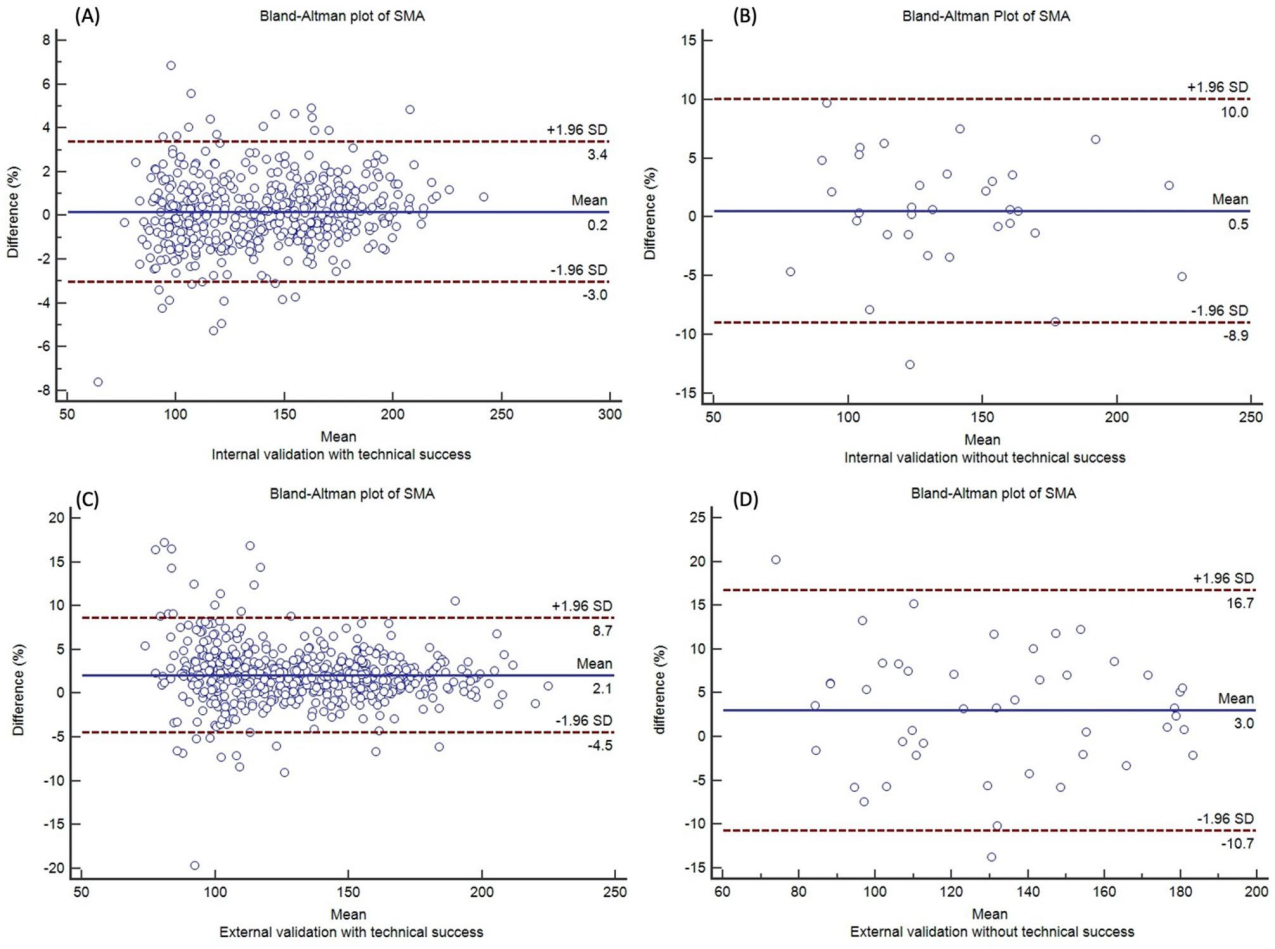
Bland Altman plots to evaluate agreement of SMA between the GT and DLM. **(A)** In subjects with technical success in the internal validation cohort, **(B)** in subjects with technical failure in the internal validation cohort, **(C)** in subjects with technical success in the external validation cohort, **(D)** in subjects with technical failure in the external validation cohort.

In both internal and external validation cohorts, the Bland–Altman plots also demonstrated that agreement of CSAs between the GT and DLM was higher for subjects with technical success than for subjects with technical failure (Fig. 6 and Supplementary Fig. 3).

The mean difference of SMA between GT and DLM-derived results ranged from 0.2 to 3.0% regardless of technical success on Bland–Altman plot. The mean difference of Sfat ranged from − 5.6 to 2.2%, and Vfat ranged from − 3.5 to 1.9%. The mean differences between GT and DLM-derived results were probably within an acceptable range of measurement variability.

The DSC values in subjects with concordant L3 levels between the GT and DLM-derived results were very high. The DSC values of SMA, Sfat, and Vfat were 0.98, 0.98, and 0.98, respectively, in the internal validation dataset and were 0.96, 0.97, and 0.97, respectively, in the external validation dataset.

### Subgroup analysis according to anatomic variation

Anatomic variation significantly influenced L3 slice selection by the DLM. The technical success rate was highest in the normal anatomy group (96.5%), followed by the thoracolumbar variation (82.6%), lumbosacral variation (63.9%), numeric variation (54.5%), and combined variation (40%) subgroups. The mean distance differences were 2.6, 7.4, 13.4, 16.5, and 21.4 mm for the normal anatomy, thoracolumbar variation, lumbosacral variation, numeric variation, and combined variation groups, respectively.

Regarding the CSA errors, anatomic variation significantly influenced Sfat and Vfat measurement, with CSA error higher than 5%, while less significantly influenced SMA measurement, with CSA error less than 5% (Table 3). Specifically, the average CSA errors between GT and DLM-derived results were 2.22% in normal anatomy subgroup and ranged from 2.37% to 4.06% in subgroups with anatomic variations.

**Table 3:** Subgroup analysis according to spine anatomy.

Subgroup	Distance difference (mm)	Technical success (%)	CSA error (%)			Bland–Altman (mean ± limits of agreement)		
			SMA	Sfat	Vfat	SMA	Sfat	Vfat
Normal anatomy (n = 943)	2.6 ± 6.0	96.5	2.22 ± 2.46	3.46 ± 4.78	3.57 ± 5.58	1.68 ± 7.22	-2.29 ± 13.13	-0.84 ± 10.03
Thoracolumbar variation (n = 46)	7.4 ± 11.9	82.6	2.73 ± 2.24	5.83 ± 8.79	5.87 ± 7.04	2.23 ± 7.90	2.41 ± 34.33	-2.69 ± 24.10
Lumbosacral variation (n = 72)	13.4 ± 15.2	63.9	3.04 ± 2.49	8.72 ± 10.63	7.94 ± 9.23	1.40 ± 10.56	2.17 ± 35.93	0.86 ± 24.84
Numeric variation (n = 11)	16.5 ± 16.1	54.5	2.37 ± 2.11	10.87 ± 7.62	10.36 ± 10.19	-0.22 ± 7.10	-3.93 ± 46.02	-1.82 ± 26.79
Combined variation (n = 10)	21.4 ± 17.0	40	4.06 ± 2.92	11.86 ± 12.66	14.95 ± 17.03	-2.53 ± 14.78	-7.15 ± 58.06	10.82 ± 67.40

*CSA* cross-sectional area, *Sfat* subcutaneous fat area, *SMA* skeletal muscle area, *Vfat* visceral fat area.

## Discussion

We were able to develop the L3SEG-net, a fully automatic DLM for selecting axial CT slice at L3 vertebral level and segmenting abdominal muscle area in an end-to-end manner. The L3SEG-net can process approximately 1,000 abdominal CT scans per day, equivalent to the 30 s/scan, in a setting of Intel^®^ CoreTM i7-7700 K GPU (8 M Cache, 4.20 GHz, Santa Clara, CA, USA), including import of CT scan data from storage database, whole APCT loading, image converting to MIP, L3 level CT slice selection, and body composition segmentation with the developed algorithm. Thus, the L3SEG-net can be helpful to perform large-scale researches^35^.

There are several unique characteristics in the L3SEG-net. First, the L3SEGnet is composed of two algorithms running sequentially as one process: a YOLOv3-based L3 slice selection algorithm and a FCN-based segmentation algorithm. When we upload one or multiple series of full abdominal CT images in the L3SEG-net, it automatically selects L3 slice CT images, segments muscle and fat areas, and provides color maps with measurement values.

Second, we trained the L3SEG-net for L3 slice selection with accurate information of anatomic variations. To identify the anatomic variations accurately, we obtained chest CT and abdominal CT scans in almost all training and validation cases and counted number of all thoracolumbar spines and ribs. Thus, the L3SEG-net is a unique model which can spotting L3 slice level with consideration of anatomic variations. Nevertheless, the normal anatomy group yielded much higher technical success rates than the anatomic variant group in the internal (96.6% vs. 67.2%) and external (96.2% vs. 67.9%) validation datasets. Among the abnormal variant subtypes, the thoracolumbar junction variant subgroup yielded similar performances to the normal anatomy group, whereas the lumbosacral junction variant subgroup and other numeric variant subgroup yielded lower technical success rates. The lower technical success of the lumbosacral junction variant subgroup may be attributable to our training process component to make the algorithm assume the iliac create as the L4 level^36^. In near the future, we will keep training the L3SEG-net for automatic spine labeling using further data.

Third, we demonstrated that the L3SEG-net’s overall segmentation accuracy of muscle areas is accurate regardless of anatomic variation in both internal and external validation cohorts. We used CSA error as a representative value of segmentation accuracy, instead of DSC. DSC evaluation was limited on the group that showed the same CT slice of GT and L3SEG-net selection. Then DSC value can present only accuracy of segmentation algorithm. Thus we suggested CSA error as an indicator reflecting accuracies of both L3 selection algorithm and segmentation algorithm, regarding clinical impact. The average CSA errors between the GT and DLM-derived results were 2.22% in normal anatomy subgroup and ranged from 2.37 to 4.06% in subgroups with anatomic variations. These results may be attributable that the distance difference between GT and DLM was less than the height of a vertebral body, as the maximum distance difference was 40 mm. According to a recent study, the muscle area measurements were similar between the L2 inferior endplate level and L4 inferior endplate level^22^.

Overall segmentation accuracy of SMA was consistent regardless of CT parameters or machine. The results were reported in prior study^37^. Various CT machines and parameters from four other hospitals were used in this study, but only portal phase abdominal CT scans were used for the analysis. The segmentation accuracy was consistent measuring SMA, Vfat, and Sfat.

There have been two prior studies that reported performance of automatic L3 level slice selection models. However, these studies did not consider the anatomic variations in the training and validation process. Belharbi et al.^24^ compared the performances of various convolutional neural networks (CNNs) for L3 slice selection with a dataset of 642 CTs of a single institution. The mean distance difference was 1.8 to 10.5 CT slices, equivalent to 3.6 to 50.5 mm. This study was limited to the task of L3 slice selection and did not have segmentation algorithm. Bridge et al.^23^ reported deep learning models for the L3 slice selection and automatic segmentation, developed based on a training cohort (n = 595) and a testing cohort (n = 534). The mean localization error was 9.4 mm. Compared to these two prior studies, our L3SEG-net showed higher accuracy in L3 slice selection.

The accuracy of body composition segmentation using L3SEG-net was comparable with prior study^14^. Park et al. reported DSC values of segmentation algorithm as 0.96, 0.97, and 0.97 (SMA, Sfat, and Vfat, respectively) in internal validation dataset and 0.97, 0.97, and 0.97 (SMA, Sfat, and Vfat, respectively) in external validation dataset. The DSC values of the L3SEG-net were slightly higher than the prior study in internal validation dataset showing 0.98, 0.98, and 0.98 (SMA, Sfat, and Vfat, respectively). The DSC value was comparable in external validation dataset showing 0.96, 0.97, and 0.97 (SMA, Sfat, and Vfat, respectively). The DSC value would be overestimated in L3SEG-net because the value was calculated in the subgroup with the identical CT slice selected both by ground truth and YOLOv3 based L3 selection algorithm.

Zopfs et al.^38^ also developed an automatic body composition analysis software tool kit using dual-energy CT. Body composition analysis was evaluated using five equidistant images from top to bottom without selection process of a proper CT slice for analysis. They reported good intra-individual consistency and good correlation of results with bioelectrical impedance analysis (BIA), which is widely used for body composition analysis due to its non-invasive nature and accessibility. But only intra-individual validation with a small subject number was available without external validation dataset.

Our study had some limitations. First, we used a relatively small size of data for training and validation of L3SEG-net deep learning model. Thus, we plan to develop a sustainable training system and keep training our L3SEG-net model using prospectively collecting CT images. Second, healthy subjects were only included for the internal and external validation cohorts. The performance of the developed DLM may require validation with large samples of patients with various diseases.

## Conclusion

In conclusion, our new deep learning model, L3SEG-net, was developed for the selection of an axial CT slice at the L3 vertebral level and the segmentation of abdominal muscle areas in an end-to-end manner. The L3SEG-net performed well regardless of anatomic variations with high L3 slice selection accuracy and segmentation accuracy. The L3SEG-net will be open for non-profit research as a web-based toolkit with hope that it can help large scale sarcopenia research.

## Supplementary Information

Supplementary Information.

## Abbreviations

CNN: Convolutional neural network
CT: Computed tomography
DSC: Dice similarity coefficient
CSA: Cross-sectional area
DLM: Deep learning model
FCN: Fully convolutional network
GT: Ground truth
MIP: Maximum intensity projection
Sfat: Subcutaneous fat area
SMA: Skeletal muscle area
Vfat: Visceral fat area
