# Supplementary material for: Development of a fully automatic deep learning system for L3 selection and body composition assessment on computed tomography
Source: Sci Rep. 2021 Nov 4;11:21656. doi: 10.1038/s41598-021-00161-5 (PMC8568923; doi:10.1038/s41598-021-00161-5)
Supplement: Supplementary file 1 — Supplementary Information. [file 41598_2021_161_MOESM1_ESM.docx]

**Supplementary Table 1. The summarization of detailed specifications used for abdominal CT acquisition.**

| **CT vendors** | **Model name** | **Filter type** | **Convolution kernel** | **Training set** | **Internal validation set** | **External validation set** |
| --- | --- | --- | --- | --- | --- | --- |
| Siemens | Definition | 0 | B30f | 9 |  |  |
|  | Definition AS | 0 | B30f | 5 |  |  |
|  | Definition AS + | 0 | B30f | 38 |  |  |
|  | Emotion | 0 | B40s | 1 |  |  |
|  | Emotion 6 | 1 | B30s | 1 |  |  |
|  | Emotion 16 | 1 | B41s | 1 |  |  |
|  | Sensation 16 | 0 | B30f, B31f | 272 | 2 | 142 |
|  | Sensation 64 | 0 | B31f | 1 |  |  |
|  | SOMATOM Definition | FLAT | B30f | 18 | 21 |  |
|  | SOMATOM Definition AS | FLAT | B30f, B40f, I30f | 49 |  |  |
|  | SOMATOM Definition AS+ | FLAT | B30f, I30f, I40f | 410 |  | 1 |
|  | SOMATOM Definition Flash | WEDGE, WEDGE_3 | B30f, I40f | 24 | 9 | 9 |
|  | SOMATOM Definition Edge | FLAT | B30f, I30f | 70 |  |  |
|  | SOMATOM PLUS 4 | N/A | AB40, AB50 | 2 |  |  |
|  | Volume Zoom | 0 | B40f | 1 |  |  |
| **Siemens_sum** |  |  |  | **902** | **32** | **152** |
| GE | BrightSpeed | BODY FILTER | SOFT | 1 |  |  |
|  | Discovery CT750 HD | BODY FILTER | STANDARD | 51 | 97 |  |
|  | HiSpeed | N/A | STANDARD | 1 |  |  |
|  | HiSpeed CT/i | LARGE BOWTIE FILTER | STANDARD | 20 |  |  |
|  | LightSpeedn Plus | BODY FILTER | STANDARD, SOFT | 33 |  |  |
|  | LightSpeed QX/i | BODY FILTER | STANDARD | 19 |  |  |
|  | LightSpeed VCT | BODY FILTER | STANDARD, SOFT | 145 | 337 |  |
|  | LightSpeed16 | BODY FILTER | STANDARD | 34 | 29 |  |
|  | Optima CT660 | BODY FILTER | STANDARD | 57 | 1 |  |
| **GE_sum** |  |  |  | **361** | **464** |  |
| Philips | Brilliance 64 | C | C |  |  | 10 |
|  | iCT 256 | A,YA | A,YA | 12 |  | 50 |
|  | Ingenuity Core 128 | B | B |  |  | 126 |
|  | Ingenuity CT | YA | YA |  |  | 194 |
|  |  |  |  |  |  |  |
| **Philips_sum** |  |  |  | **12** |  | **380** |
| Hitachi | Presto | N/A | 4 | 1 |  |  |
| TOSHIBA | Aquilion PRIME |  |  |  |  | 54 |
|  | Aquilion | LARGE,EC | FC 13, FC 08, FC 04, FC 18 | 10 |  |  |
| **Others_sum** |  |  |  | **11** |  | **54** |
| **Sum** |  |  |  | **1286** | **496** | **586** |

**Supplementary figures**


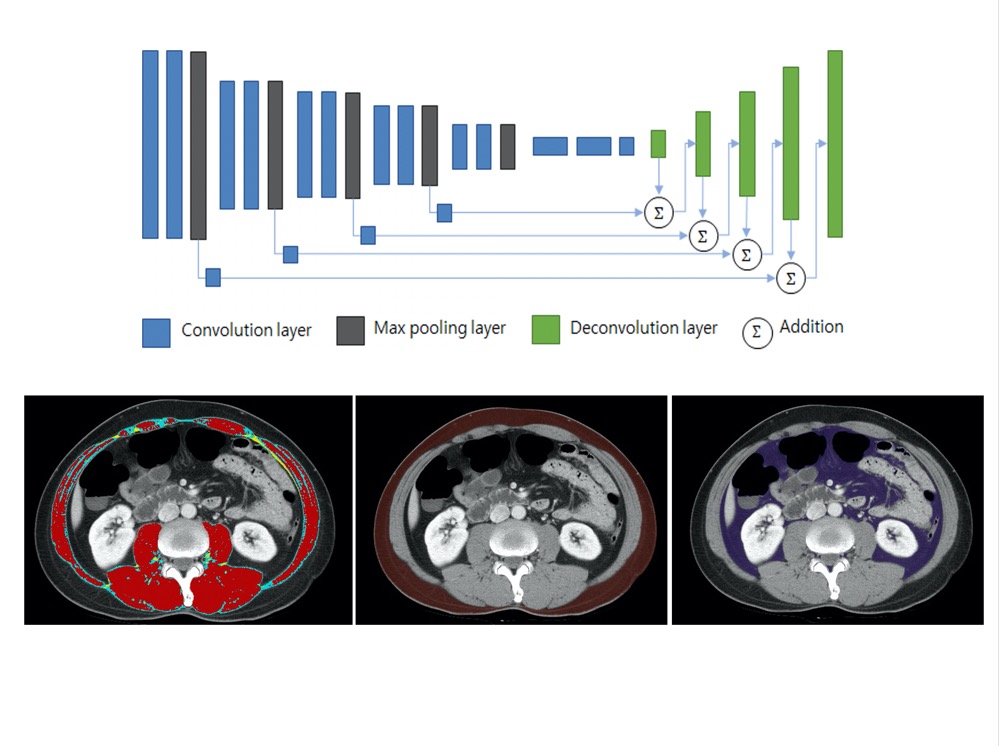


Supplementary Figure 1. The network architecture of a fully convolutional network–based segmentation model. Post-processing based on Hounsfield units (HU) was added to separate the intramuscular adipose tissue from skeletal muscle area. Red and blue areas indicate skeletal muscle area (−29 to 150 HU) and area in yellow indicates intramuscular adipose tissue (−190 to −30 HU). Brown area indicates subcutaneous fat area and area in purple represents visceral fat area.


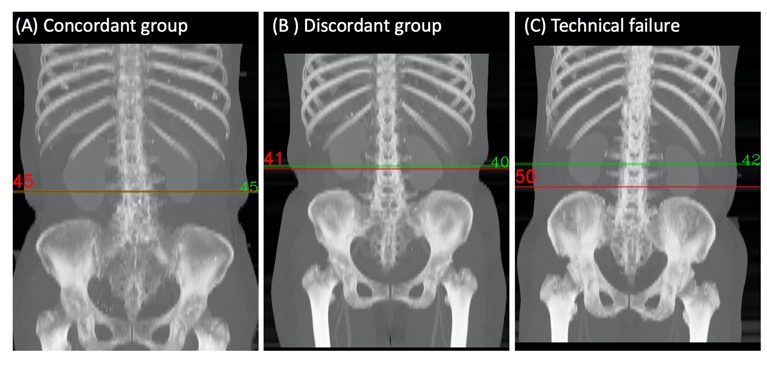


Supplementary Figure 2. Examples of technical success and technical failure.

(A) Technical success when L3 CT slice numbers are identical between the ground truth (GT) and the deep learning model (DLM)–derived results.

(B) Technical success when the distance difference between the GT and the DLM-derived results is less than 10 mm.

(C) Technical failure when the distance difference between the GT and the DLM-derived results is greater than 10 mm.


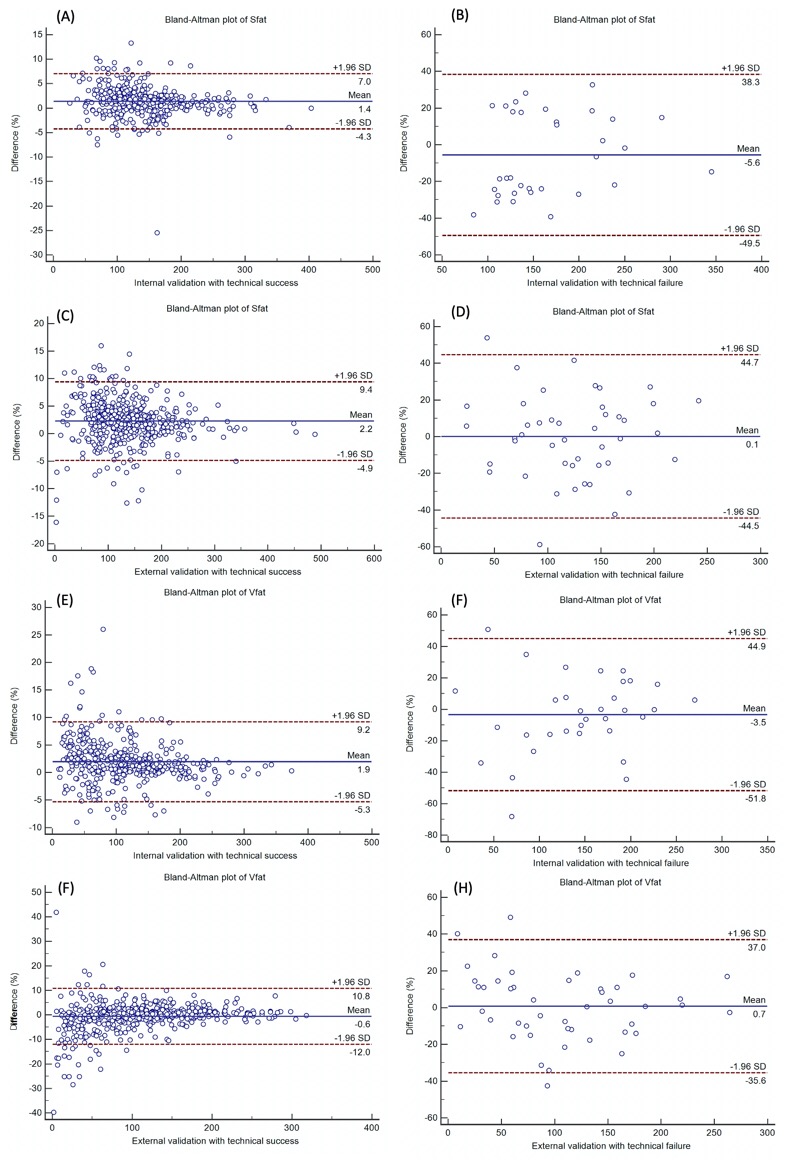


Supplementary Figure 3. Bland Altman plots to evaluate agreement of Sfat and Vfat between the GT and DLM

(A) Sfat in subjects with technical success in the internal validation cohort

(B) Sfat in subjects with technical failure in the internal validation cohort

(C) Sfat in subjects with technical success in the external validation cohort

(D) Sfat in subjects with technical failure in the external validation cohort

(E) Vfat in subjects with technical success in the internal validation cohort

(F) Vfat in subjects with technical failure in the internal validation cohort

(G) Vfat in subjects with technical success in the external validation cohort

(H) Vfat in subjects with technical failure in the external validation cohort
